# Effect of germanium oxide on the structural aspects and bioactivity of bioactive silicate glass

**DOI:** 10.1038/s41598-023-36649-5

**Published:** 2023-06-13

**Authors:** Taha M. Tiama, Medhat A. Ibrahim, Mohamed H. Sharaf, Ahmed F. Mabied

**Affiliations:** 1 Department of Basic Sciences, October High Institute of Engineering & Technology-OHI, 6th of October City, Giza, Egypt; 2 Molecular Spectroscopy and Modeling Unit, Spectroscopy Department, National Research Centre, 33 El-Bohouth St., Dokki, Giza, 12622 Egypt; 3 Botany and Microbiology Department, Faculty of Science, Al-Azhar University, Cairo, Egypt; 4 X-Ray Crystallography Lab., Solid State Physics Department, National Research Centre, 33 El-Bohouth St., Dokki, Giza, 12622 Egypt

**Keywords:** Materials science, Physics

## Abstract

Ternary silicate glass (69SiO_2_–27CaO–4P_2_O_5_) was synthesized with the sol–gel route, and different percentages of germanium oxide GeO_2_ (6.25, 12.5, and 25%) and polyacrylic acid (PAA) were added. DFT calculations were performed at the B3LYP/LanL2DZ level of theory for molecular modelling. X-ray powder diffraction (XRPD) was used to study the effect of GeO_2_/PAA on the structural properties. The samples were further characterized using DSC, ART-FTIR, and mechanical tests. Bioactivity and antibacterial tests were assessed to trace the influence of GeO_2_ on biocompatibility with biological systems. Modelling results demonstrate that molecular electrostatic potential (MESP) indicated an enhancement of the electronegativity of the studied models. While both the total dipole moment and HOMO/LUMO energy reflect the increased reactivity of the P_4_O_10_ molecule. XRPD results confirmed the samples formation and revealed the correlation between the crystallinity and the properties, showing that crystalline hydroxyapatite (HA) is clearly formed in the highest percentages of GeO_2_, proposing 25% as a strong candidate for medical applications, consistent with the results of mechanical properties and the rest of the characterization results. Simulated body fluid (SBF) in vitro experiments showed promising biocompatibility. The samples showed remarkable antimicrobial and bioactivity, with the strongest effect at 25%. The experimental findings of this study revealed that the incorporation of GeO2 into the glass in terms of structural characteristics, bioactivity, antimicrobial properties, and mechanical properties is advantageous for biomedical fields and especially for dental applications.

## Introduction

Bioactive glass is composed of three-dimensional networks of silicate/phosphate, most of them are based on the Na_2_O, CaO, P_2_O_5_, and SiO_2_ could be implemented in biological system forming strong chemical bonds with bones^1,2^. It is dedicated among other biomaterial-driven for different biomedical applications^2,3^. Recently, bioglass cement (BGC) used to fill voids and fractures installation, due to its adhesive nature, radio-opacity and durability. Through the acid–base reaction between glass ionomer and aqueous Poly Acrylic Acid (PAA), it can chemically bind to bone^4^. Also, besides their ability to chemically bind to bones, but they are fragile in tension, which reduces their use in stabilizing a pregnant fracture^5^. BGC has been used in both ear, nose and throat (ENT) and dental applications^6–11^. The advantages of using BGC in dentistry are many including biocompatibility, bioactivity, high dimensional stability, good resistance to cohesive failure, negligible shrinkage upon installation. Efforts have been made to improve these materials and use them for biomedical applications^12–14^. Germanium oxide is an inorganic compound that may have a major role when used in BGC to improve their properties, it has the theoretical ability to replace Si in the glass network^15,16^. From previous research, it was reported that, the GeO_2_ was incorporated into borate-based glass ionomer (BGG)^17,18^. it was found that there is an increase in the setting and working time (handling properties) formulated from these glasses as a result of incorporating Ge, as this reduces the number of non-bridged oxygen’s (NBOs) in the glass lattice resulting in reduced setup and working times^19^. Dickey et al.^20^ mentioned Ge-based ionic glasses and have not succeeded in evaluating whether this cement was injectable or not for use in spinal stabilization. On the other side, Molecular modeling at different levels of theory could be utilized to fulfill the experimental efforts elucidating the molecular structures of many systems and compounds. In this sense, glass models were generated using MD simulations to investigate different factors mimicking the activity cations for elucidating physical and chemical properties^21^. Another approach was utilized via ab initio route to investigate diverse glass physical parameters^22^. DFT was utilized to investigate structure-performance relationship for polypropylene composites modified with reduced graphene oxide rGO^23^. It is stated that, the DFT was successfully employed to calculate the activation energy. It is also stated that, computational methods could be applied to investigate physical, chemical and biological interactions for both glass as well as hydroxyapatite. It was utilized with other characterizing tools to elucidate the effect of physical parameters such as temperature on in vitro bioactivity, molecular and mechanical properties of hydroxyapatite^24–26^. Based upon the biological activity and unique molecular properties, it was subsequently dedicated such classes of compounds for various biomedical applications^27,28^.

Identifying the structural properties of materials using X-ray diffraction has a great impact on improving their properties and maximizing their utilization in various applications, which is of great importance in studying the crystalline phases of glass materials^29,30^.

Based on the aforementioned the aim of the present work is to develop bioactive glass more combatable with biological systems and antimicrobial activity specially for dental applications. Silicate glass (69SiO_2_–27CaO–4P_2_O_5_) system will be prepared and modified with germanium oxide/poly acrylic acid (GeO_2_/PAA). To understand the molecular interaction of the studied samples calculations based on DFT:B3LYP/LanL2DZ will performed to calculate some physical parameters such TDM, ∆E then mapping MESP is conducted to indicate the active sites of the studied structures. XRPD study will executed to reveal the effect of GeO_2_/PAA on the structural characteristics and the associated changes in the physical and biological properties.

## Materials and methods

### Calculation details

Model structures simulated the interaction of P_4_O_10_Si_3_O_6_ with CaO and GeO_2_ using Gaussian 09 package^31^ at Molecular Spectroscopy and Modeling unite, National Research Centre, Egypt. The Model structures were optimized and calculated using DFT at B3LYP/LanL2DZ model^32–34^. Electronic properties and reactivity were studied by calculating MESP mapping for model structures^35–37^.

### Chemicals and reagents

Sigma Aldrich supplied tetraethylorthosilicate (TEOS), calcium nitrate tetrahydrate Ca (NO)3H2O, and triethyl phosphate (TEP) (98 percent), 33 percent ammonia solution, and 68 percent nitric acid, deionized water was used to dilute both the nitric acid and ammonia solutions to 2 M (mol).

### Samples preparation

For this work, four glass samples were created: three germanium Oxide -containing glasses and a germanium-free 69SiO_2_–27CaO–4P_2_O_5_ (mol percent) glass (SCP). GeO_2_ concentrations of (SCPGe 6.25%), (SCPGe 12.5%) and (SCPGe 25%) mol% are present in the germanium-containing glasses. The Control glass was annealed at Tg for 2 h. This glass powder was employed in the characterization and bioactivity experiments described in section “Characterizing techniques”.

On a glass plate, the glass granules were thoroughly mixed with polyacrylic acid (PAA—Mw, 210,000, 90 m, Kent, UK) and sterile DI water to make cement. The cement was made with a 2:1 powder to liquid (P: L) ratio with PAA additions of 50 wt percent. Glass and PAA powders were used in the powdered component, while sterile DI water was used in the liquid component. Before insertion into the appropriate molds, the components were thoroughly mixed in the 20 s. A specially designed Teflon mold was prepared to form cylindrical samples with dimensions of 3 mm in diameter and 6 mm in height. These dimensions were determined according to (International Standards Organization (ISO) No. 9917 (2007)^38^. Then another designed Teflon mold was prepared to form disk samples with dimensions of 6 mm in diameter and 3 mm in height. These dimensions were determined according to International Standards Organization (ISO) No. 9917 (2007)^38^. Circular discs (5 mm in diameter) were immersed in 40 ml of simulated bodily fluid (SBF) solution for 0, 4, 8, and 16 days at 37 °C in vitro bioactivity tests. The membranes are cleaned with distilled water and dried after being taken from the SBF. As a result, SBF is made by dissolving NaCl, NaHCO_3_, KCl, K_2_HPO_4_·3H_2_O, MgCl_2_·6H_2_O, and Na_2_SO_4_ in distilled water and buffering with Tris and HCl solution to achieve a pH of 7.4, as described by Kokubo and Takadama^39^. Sigma Aldrich provided all of the chemicals.

## Characterizing techniques

### Differential calorimetry scanning (DSC)

Study DSC to assess the thermal stability of the phospho—silicate bioactive glass. The powder of the sample was placed in a DSC sample holder, and we have the calorimetric curves in the temperature range of 25–1200 °C were recorded at a heating rate of 10 °C min^−1^ in a nitrogen environment (30.0 ml min^−1^) on a DSC-60 system (Shimadzu, Japan).

### X-ray diffraction measurement (XRD)

X-ray diffraction study were performed at X-ray Crystallography laboratory, Physics Research Institute, National Research Center. Powder patterns of the studied samples were measured using an X-ray diffractometer (PANalytical Empyrean, Netherlands) with Cu kα- radiation at 30 mA and 45 kV in ambient temperature. The diffractograms were scanned in the 2-theta range of 10°–80°, with scan step 0.026, counting time 40 s step^−1^. A standard quartz sample was used for line profile analysis. Data analysis and visualization were performed through HighScore Plus suit^40^ and WinPLOTR^41^.

### ATR-FTIR measurement

ATR-FTIR spectral data was collected in the range 4000–400 cm^−1^ using spectrometer VERTEX 80 (Bruker Corporation, Germany) coupled with Platinum Diamond ATR, which consists of a diamond disc as an internal reflection element.

### Mechanical properties

#### Compressive strength testing

In a Teflon mould mounted on a glass plate, the combined cement was condensed. Celluloid strips were used to cover the samples, and another glass plate was used to push them down. After hardening, the samples were taken out of the mould and placed in distilled water for 24 h before being tested. The compressive strength of all samples was measured using a universal mechanical testing machine (Lloyd instrument, LRX plus PI No. 01/2962 England). At a crosshead speed of 1 mm min^−1^, the samples were loaded on the Lloyd mechanical testing equipment. Between the two metal plates, the samples were positioned vertically with a flat end. The load was applied until the sample was crushed, and the peak force necessary to fracture each sample in Newton was recorded from the load. The compressive strength was calculated in (MPa) using the following equation:

$${\text{CS }} = {\text{ 4P}}/\pi {\text{d}}^{{2}}$$

where (CS) is the compressive strength (MPa), (P) is the load at the fracture point (N), (d) is the diameter (mm) of the sample, and (π) is a constant = 3.14.

#### Diametral tensile strength testing

Each group was prepared as described before for the compressive strength test. This is a different way to test brittle materials that can break at the grips during testing. Compressive testing is used to assess a fragile material's ultimate tensile strength. The disc samples were mounted on the Lloyd mechanical testing equipment, and the load was applied to the samples with a crosshead speed of 0.5 mm min^−1^, compressing the samples until fracture occurred. Using the following equation, the diametral tensile strength was computed in (MPa):

$${\text{DTS }} = {\text{ 2P}}/\pi {\text{dt}}$$

where (DTS) is the diametral tensile strength (MPa), (P) is the load (N) at the fracture point, (d) is the diameter (mm) of the samples, (t) is the height (mm) of the samples and (π) is a constant = 3.14.

#### Shear bond strength testing

Using a customised Teflon mould, cylindrical samples of glass ionomer cement were created on the flat dentine surface in this study (5 mm length x 2 mm diameter). These dimensions were obtained according to International Standards Organization (ISO) No. 9917. (2007)^38^. The mould is then reassembled. A material testing machine with a cross-head speed of 1/2 mm per minute was used to assess shear bond strength. The bond strength will be evaluated using a circular interface shear bond strength setup.

### Antibacterial activities for GeO_2_/Bioglass

#### Antimicrobial activity

The antimicrobial activity of SCPGe was evaluated on Muller Hinton agar (MHA, India) for bacteria and PDA for *Candida albicans*. Twenty-four hours’ young culture of *Staphylococcus aureus* ATCC 6538, *Bacillus subtilis* ATCC 6633, *Escherichia coli* ATCC 8739, *Salmonella typhimurium* ATCC14028 and *Klebsiella pneumonia* ATCC 13,883 and 48 h young culture of *Candida albicans* ATCC10231 were cultured on the surface of prepared MHA and PDA for bacteria and *Candida albicans*, respectively. Wells (6 mm) were cut using a sterile corn borer; 100 µl of SCPGe was transferred to the well and left for 2 h at 4 ℃. Rifampin was used as a control for bacterial strain, while fluconazole was used as a control for *Candida albicans*, and then, the plates were incubated for 24 h at 37 ℃ for bacteria and 48 h at 28 ℃ for *Candida albicans*. After incubation, the inhibition zones were measured and recorded and this experiment was repeated three times^38,42^.

#### Determination of minimum inhibitory concentration (MIC)

MIC values of different SCPGe Concentration (from 25 to 0.781%) were performed against *Staphylococcus aureus* ATCC 6538, *Bacillus subtilis* ATCC 6633, *Escherichia coli* ATCC 8739, *Salmonella typhimurium* ATCC14028 and *Klebsiella pneumonia* ATCC 13,883 and 48 h young culture of *Candida albicans* ATCC10231 and checked by broth microdilution assay^43^. Different concentrations of SCPGe were prepared. Test sample (100 µl) of various concentrations was added into sterile microtiter plate wells filled with 100 µl of double strength Mueller Hinton (MH) broth to make final concentrations at 25, 12.5, 6.25, 3.125, 1.56 and 0.781%. Bacterial cell suspension (50 µl) corresponding to (OD equivalent to 0.5 McFarland standard) was added in all wells except those in negative control well. Positive Control wells were filled with MH broth and bacterial suspension to check for adequacy of the broth to support bacteria growth. The negative control wells were consisted of sterile distilled water and Mueller Hinton broth to check sterility. The plates were incubated at 37 °C for 24 h. To indicate bacterial growth, 30 µl of resazurin solution (0.02% wt./v) (HiMedia) was added to each well, and the plate was pre-incubated overnight. A change in color from blue to purple, red, or pink indicated the growth of bacteria. A change in color of growth control wells to pink, red or purple indicated the proper growth of the isolate and no change in color of sterile control well-indicated absence of contaminants. The experiment was performed in duplicates and repeated three times^43,44^.

### Cytotoxic activity

Cell culture cultivation HFB4 (human normal fibroblast) was obtained from Science Way for scientific researches (Nasr city, Egypt) and used for the cytotoxicity assay. The tested cell was maintained in Dulbecco’s Modified Eagle Medium supplemented with 100 mg ml^−1^ streptomycin, 100 units ml^−1^ penicillin, and 10% heat inactivated fetal bovine serum in a humidified, 5% (v/v) CO_2_ atmosphere at 37 °C. The percentage of cell viability was calculated using the following formula:

$${\text{Viability }}\% = \, \left( {\text{Test OD}} \right)/\left( {\text{Control OD}} \right) \, \times { 1}00$$

#### MTT cytotoxicity assay

Aliquots of 50 μl cell suspension (3 × 103 cells) were seeded in 96-well microplates and incubated in complete media for 24 h. Then, cells were treated for 48 h with another aliquot of 50 μl media containing the Germanium/bioglass (dissolved in 0.5% DMSO) at different concentrations (1000–31.25 μg ml^−1^). The plates were incubated at 37 °C and 5% CO2 atmospheric conditions for 24 h. The cells were incubated with 50 μl well^−1^ of (3-(4,5-dimethylthiazol-2-yl)-2,5-diphenyltetrazolium bromide (MTT) solution. The absorbance of each well was read at a wavelength of 560 nm using an ELISA reader^45^.

## Results and discussions

### Molecular modeling

**Figure 1:**
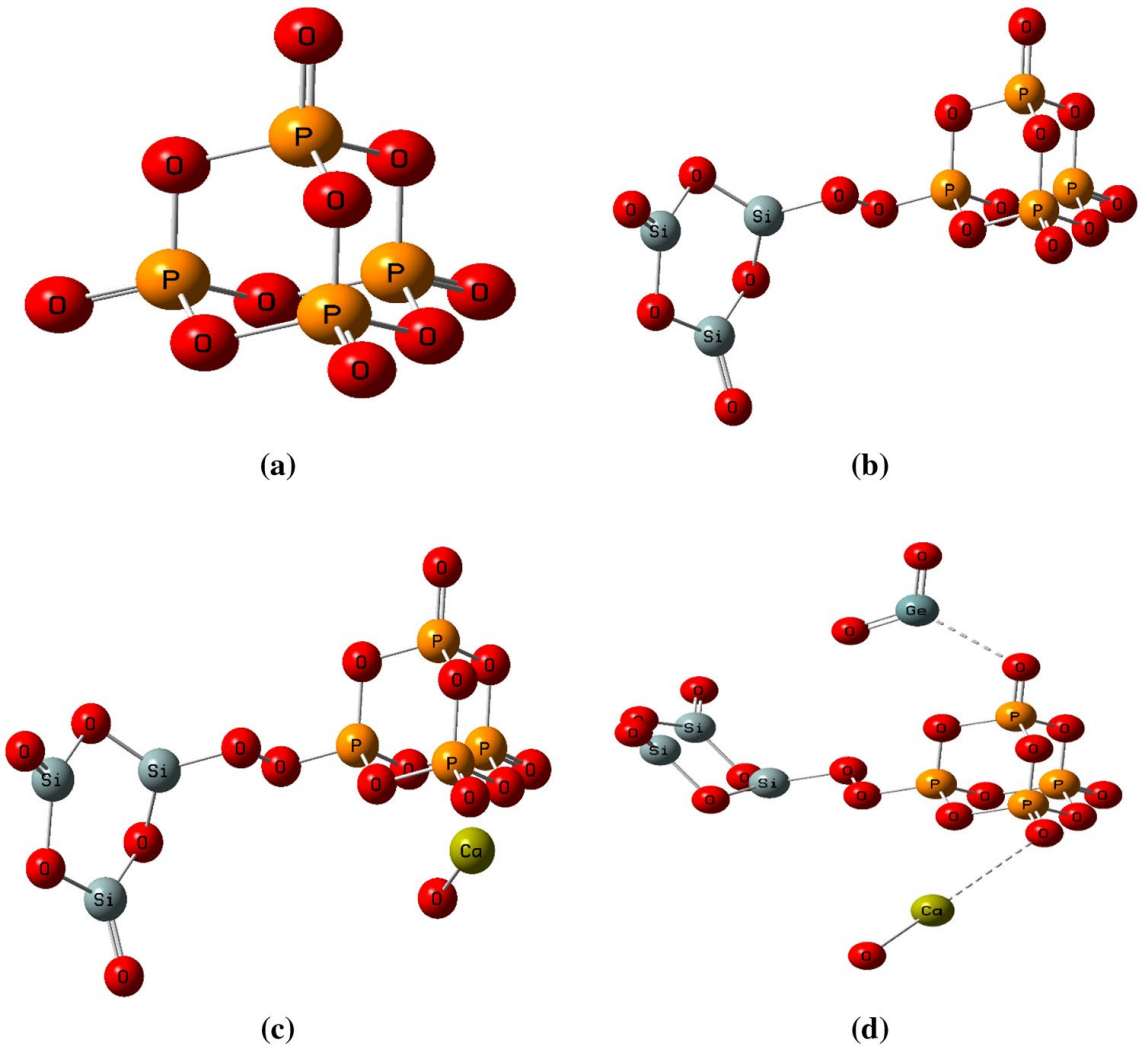
Optimized structures of (**a**) P_4_O_10_, (**b**) P_4_O_10_ doped with Si_3_O_6_, (**c**) P_4_O_10_–Si_3_O_6_ doped with CaO and (**d**) P_4_O_10_–Si_3_O_6_–CaO doped with GeO_2_.

The first step is to describe how the model molecules were built. As indicated in Fig. 1 there four model molecules. Figure 1a indicated the main model molecule which is P_4_O_10_. Figure 1b, indicated P_4_O_10_ doped with Si_3_O_6_. Figure 1c show the same model P_4_O_10_–Si_3_O_6__doped_ with CaO and finally Fig. 1d show P_4_O_10_–Si_3_O_6_–CaO doped with GeO_2_. For each model the energy optimization is calculated then both total dipole moment and molecular electrostatic potential are calculated at DFT: B3LYP/LanL2DZ level of theory.

#### TDM and HOMO/LUMO bandgap energy

The optimized structures of phosphate glass which is composed of P_4_O_10_ and P_4_O_10_ doped with Si_3_O_6_, CaO and GeO_2_ are presented in Fig. 1. Table 1 provides the computed total dipole moment (TDM) and HOMO/LUMO bandgap energy (ΔE) for P_4_O_10_, P_4_O_10_ doped with Si_3_O_6_, model of P_4_O_10_–Si_3_O_6_ doped with CaO and P_4_O_10_–Si_3_O_6_–CaO model molecule doped with GeO_2_ which are computed at B3LYP/LanL2DZ theoretical level. TDM of P_4_O_10_ which is 0.000 Debye increased because of doping with Si_3_O_6_ to 5.080 Debye. However, those for P_4_O_10_–Si_3_O_6_ model molecule doped with CaO increased to 11.938 Debye and 16.272 Debye for P_4_O_10_–Si_3_O_6_–CaO–GeO_2_. Based on the computed results, it is concluded that the TDM value of P_4_O_10_ increased with increasing the doping level.

**Table 1:** Calculated total dipole moment (TDM) as Debye and HOMO/LUMO bandgap energy as eV for: P_4_O_10_, P_4_O_10_ doped with Si_3_O_6_, P_4_O_10_–Si_3_O_6_ doped with CaO and P_4_O_10_–Si_3_O_6_–CaO doped with GeO_2_ at B3LYP/LanL2DZ.

Structures	TDM (Debye)	∆E (eV)
P_4_O_10_	0.000	3.623
P_4_O_10_–Si_3_O_6_	5.080	0.692
P_4_O_10_–Si_3_O_6_–CaO	11.938	0.395
P_4_O_10_–Si_3_O_6_–CaO–GeO_2_	16.272	0.376

On the other hand, the energy required to transfer an electron from the highest occupied molecular orbital (HOMO) to the lowest unoccupied molecular orbital (LUMO) in the P_4_O_10_ structure decreased from 3.623 eV to 0.692, 0.395 and 0.376 eV for P_4_O_10_ doped with Si_3_O_6_, P_4_O_10_-Si_3_O_6_ doped with CaO and P_4_O_10_–Si_3_O_6_–CaO doped with GeO_2_ respectively.

#### Molecular electrostatic potential

**Figure 2:**
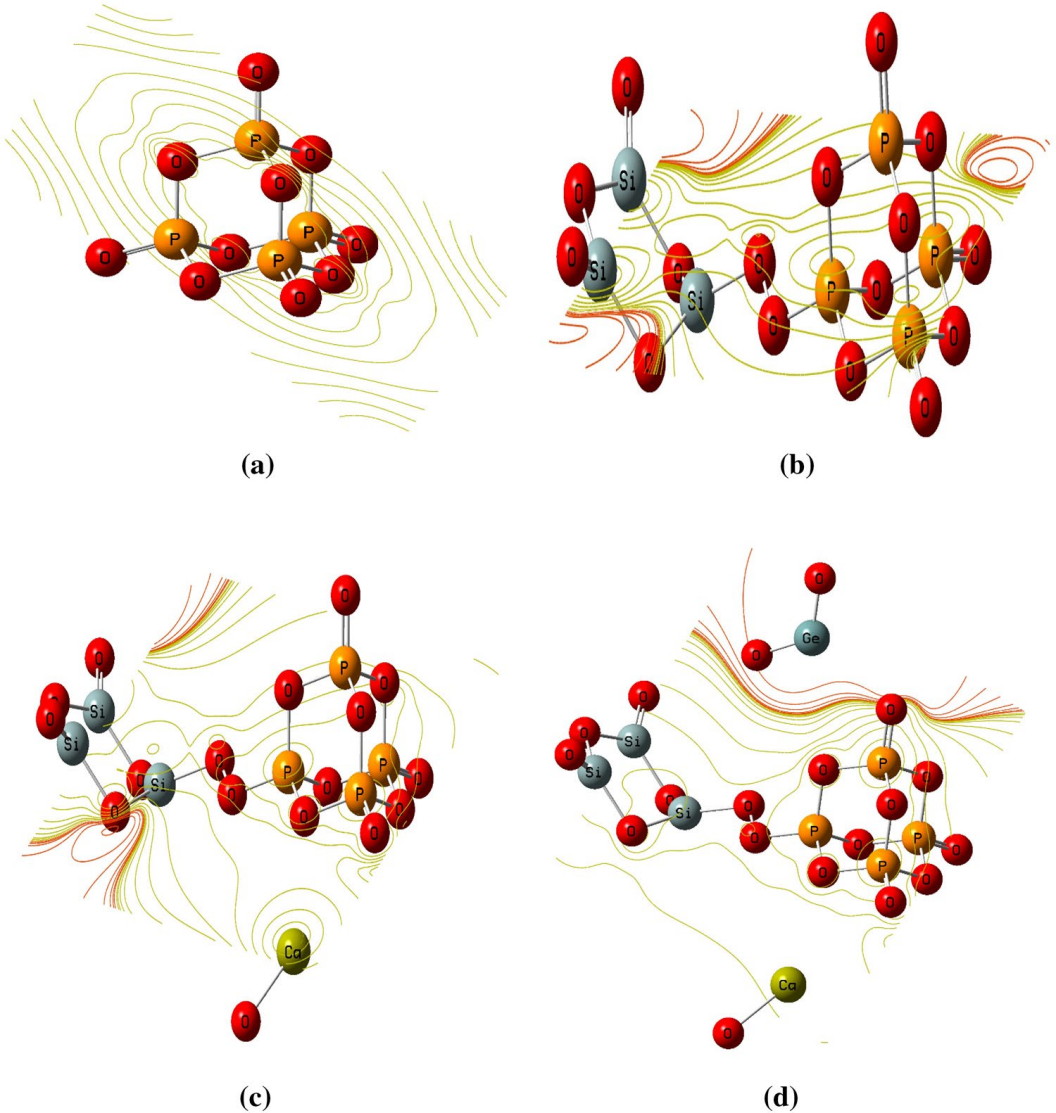
DFT calculated MESP of (**a**) P_4_O_10_, (**b**) P_4_O_10_ doped with Si_3_O_6_, (**c**) P_4_O_10_–Si_3_O_6_ doped withCaO and (**d**) P_4_O_10_–Si_3_O_6_–CaO doped with GeO_2_ at B3LYP/LanL2DZ.

Molecular electrostatic potential (MESP) maps were generated for the proposed structures as presented in Fig. 2. MESP maps are studied at the DFT theoretical level utilizing B3LYP/LanL2DZ. MESP maps generally provide a simple way of clarifying the distribution of electronic charges within the studied structure, thus providing the most likely active sites. MESP colors ranges from the most negative to the most positive in the following order: red, orange, yellow, green, cyan, and dark blue, with red corresponding to extreme negative potential and blue to positive. Also, the yellow region indicates a lower negative potential than the red, and the yellow indicates the neutral potential region^35–37^.

The calculated maps, as presented in Fig. 2, consists of only two colors which are red and yellow color representing the extreme negative region for red color and neutral sites for yellow.

The distribution of the charges of the atoms can also be related to some extent via the electronegativity of atoms to which they are attached. Atoms with high electronegativity are surrounded by red color when combined with atoms other than electronegative one. The presence of several atoms with nearly equal electronegativity narrows the color distribution considerably. Therefore, one can rely on MESP maps when determining whether an active site of interest can undergo a chemical interaction.

Regarding MESP map of pure P_4_O_10_, there is only one color within the constructed map which is the yellow color which means that P_4_O_10_ is a neutral structure (i.e., inactive) as presented in Fig. 2a. Thus, the TDM and HOMO/LUMO bandgap results are confirmed by the MESP map results. However, due to doping with Si_3_O_6_ two colors are observed: red and yellow as showed in Fig. 2b. The red color located around the Si_3_O_6_ structure and extends to some regions within P_4_O_10_. This means that the electronegativity of P_4_O_10_ increased due to doping with Si_3_O_6_. Meanwhile in Fig. 2c and d depicts the improvements in the electronegativity of P_4_O_10_–Si_3_O_6_ structure upon the interaction with CaO and P_4_O_10_–Si_3_O_6_–CaO model molecule dopped with GeO_2_ respectively. Increasing the doping level results in enhancement of the electronegativity of the studied models as the intensity of red color increased. The increment in the red color intensity upon increasing the doping level confirms the TDM results and HOMO/LUMO energy which reflects the increased reactivity of P_4_O_10_ molecule.

### Differential calorimetric analyses (DSC) and thermogravimetric analyses (TGA)

**Figure 3:**
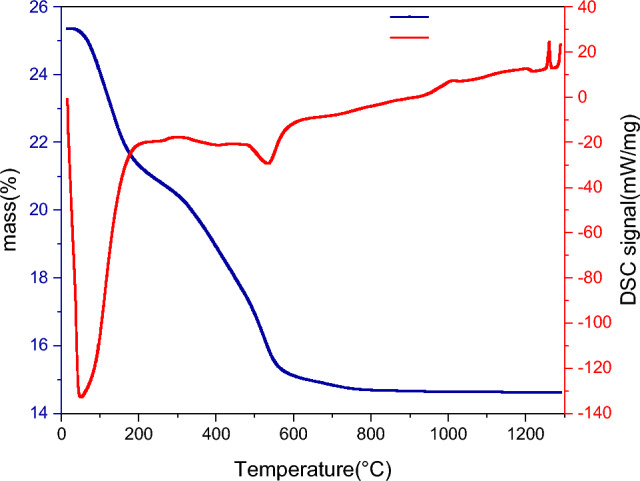
DSC and TGA curve of the bioactive glass gel powder after dray at 120 °C for 2 days.

We used DSC to test the thermal stability of SCP sample. The dried gel glass sample used to obtain the DSC curve shows in Fig. 3. In this study, the sample is heated from room temperature to 1200 °C. The Tg (characteristic transition temperatures), Tc (crystallization temperatures) and Tm (melting temperatures) for pristine condition glasses as were investigated (533 °C, 1005 °C, 1200 °C). In the DSC the first endothermic peak at 176.23 °C to drive off H_2_O and other solvent. the exothermic peak at 253.6 °C due to the pyrolysis reaction of free organic species and/or the release of the resulting water from the further condensation of silanol and P–OH groups as was described in other study^46^. The endothermic peak start at 535.27 °C is related to the departure of nitrate group. DSC test explain that all nitrates were removed by 597.53 °C^47^. The glass transition (Tg) of the gel glass started at approximately 597.07 °C. While the exothermic peak centered at 1005.24 °C, which corresponds to the crystallization process of the glass.

### XRD

**Figure 4:**
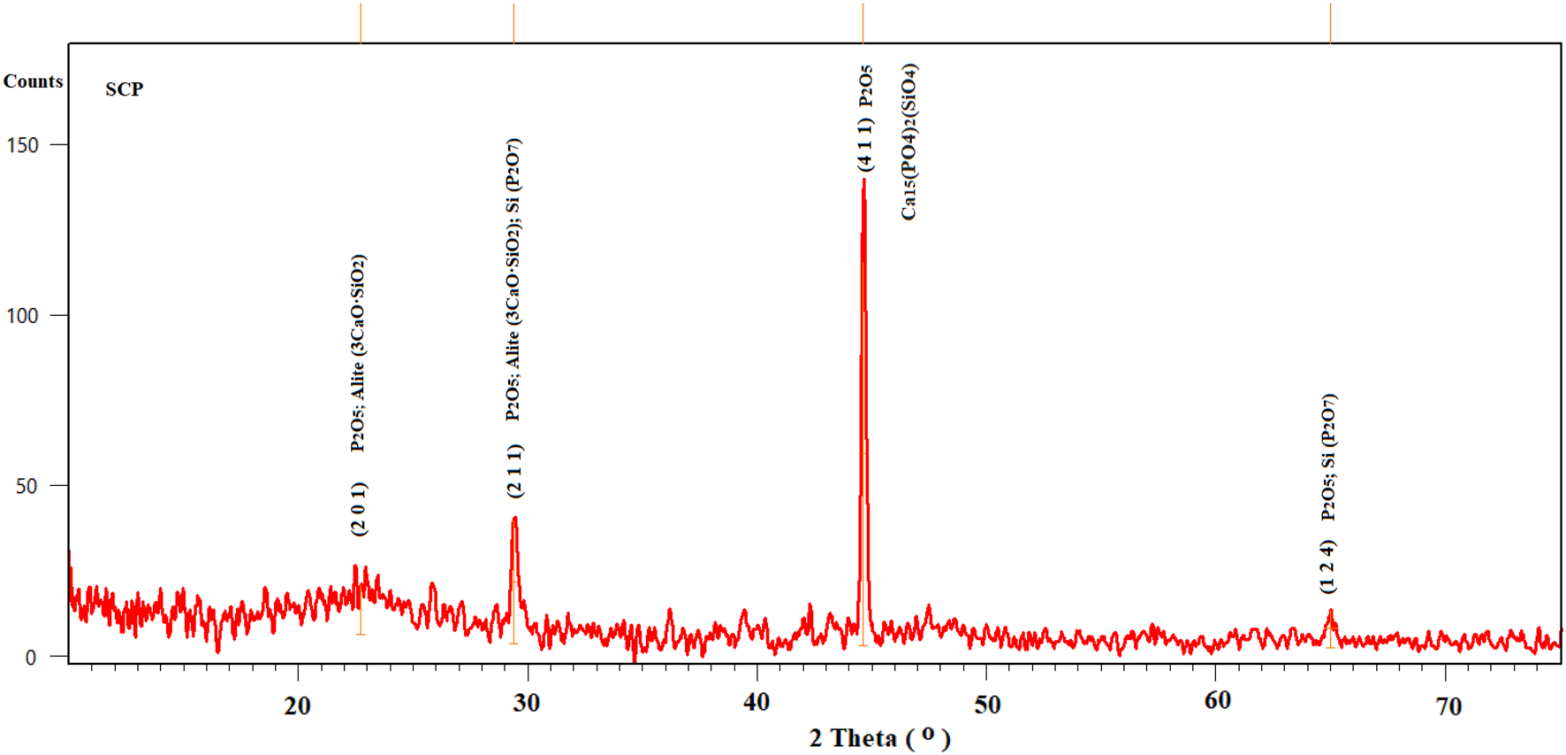
Diffraction pattern of bioactive glass (SCP) before adding GeO_2_.

**Figure 5:**
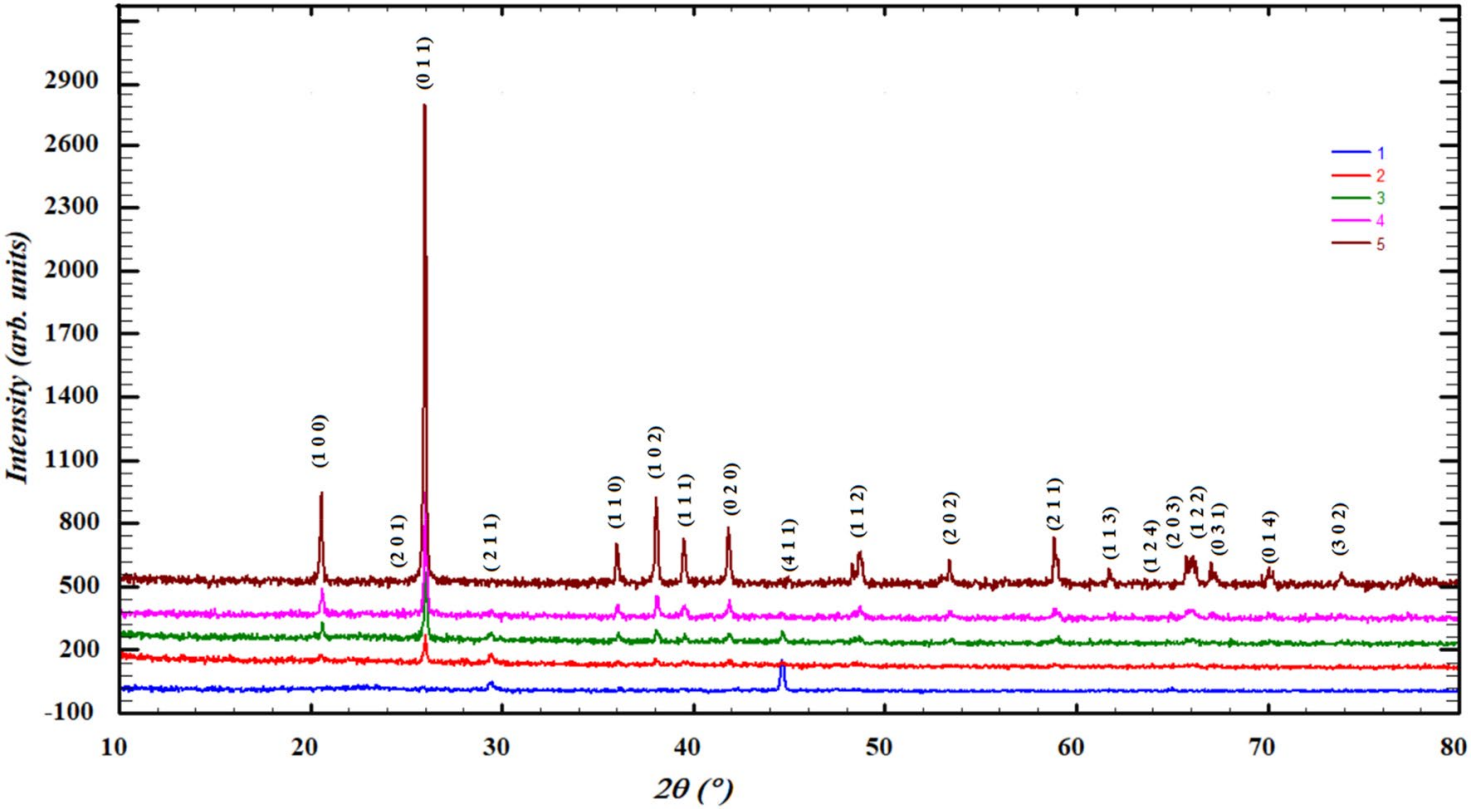
Diffraction patterns of SCP mixed with the different percentage of GeO_2_.

The change in X-ray diffraction patterns was tracked in all stages of the study for the samples. The recorded diffraction patterns revealed the presence of SCP in the crystalline form and showed the characteristic diffraction peaks of SCP and germanium oxide, as shown in the Table 2, Figs. 4 and 5. The diffraction peaks of SCP confirmed by matching the cards 96-810-3588, 96-231-0557, 96-154-0706 and 96-153-0880 of COD database (Fig. 4), also very closed to the reported similar bioglass systems^48–50^. GeO_2_ peaks matched accurately PDF2 card no 98-005-9625 for GeO_2_. It is clear from the Fig. 4 that is the most prominent crystalline peak in SCP pattern at 44.6° dedicated to P_2_O_5_, also reported for calcium phosphate silicate (Ca_15_(PO_4_)_2_(SiO_4_)_6_)^49^, which can be explained by the fact that P_2_O_5_ is the most active component of SCP system to form a crystalline nucleus of the crystalline state. It is in consent with previously published literature^51^.

**Table 2:** Bioactive glass samples with code.

Bioglass sample	Code
SCP	1
SCPGe 6.25%	2
SCPGe 12.5%	3
SCPGe 25%	4
GeO_2_	5

It is noticeable that the bioglass sample (SCP) appears in the crystalline state, although it has not been heated to the crystallization temperature. This can be explained by what has been published about the ability of bioglass to recrystallize itself at room temperature and in ambient conditions^50^. Moreover, the crystallization activity of P_2_O_5_ ions^51^.

**Figure 6:**
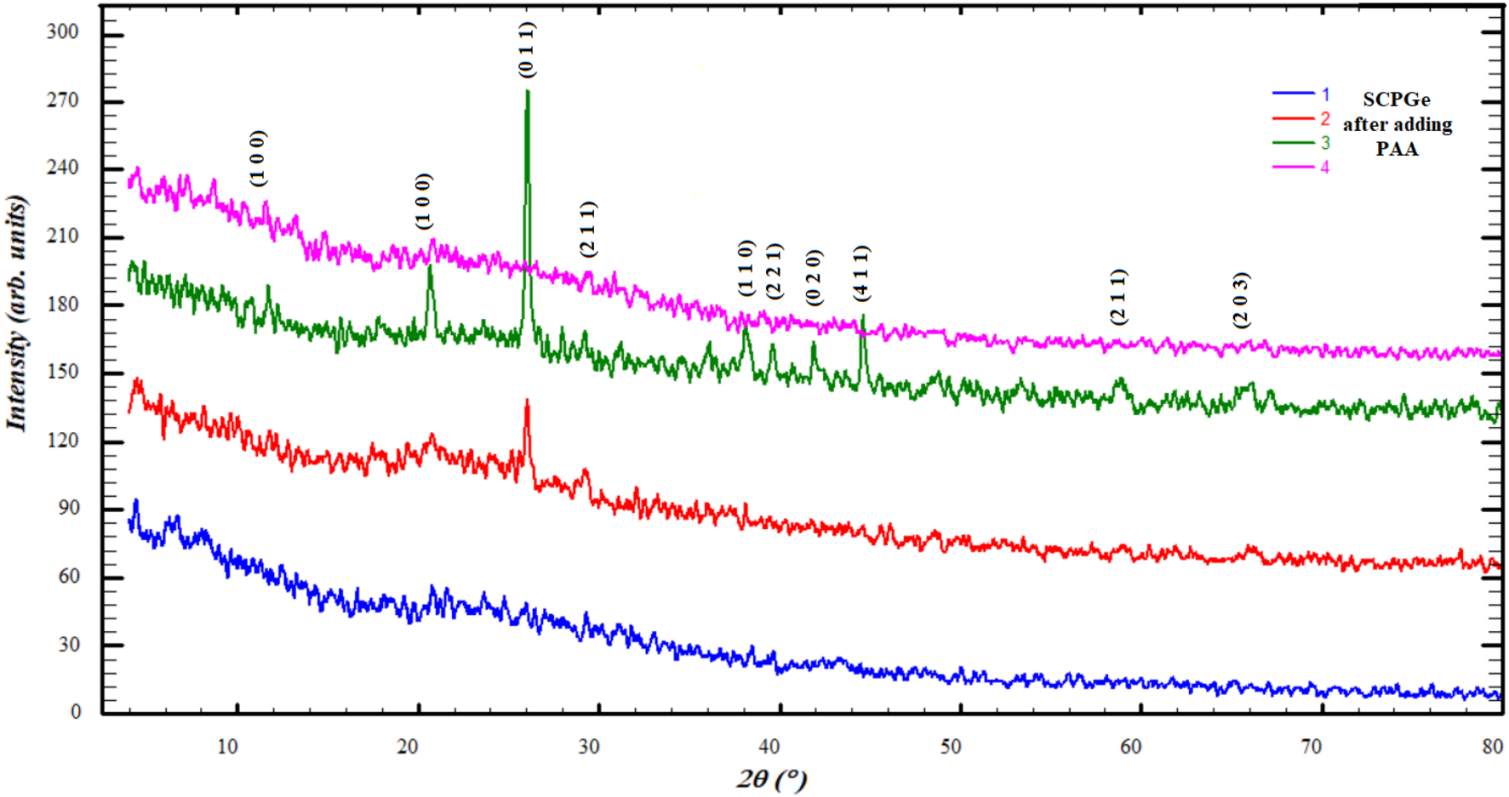
Diffraction patterns after adding the polyacrylic acid.

After the addition of polyacrylic acid (PAA), the diffraction results showed a change in the crystalline state of the samples with different percentages of germanium oxide (Fig. 6), which due to effect of the amorphous nature of the polyacrylic acid^52,53^.

The disappearance of the crystallinity in the bioglass sample 1 (Fig. 6) may be attributed to the process of recrystallization of the samples after dissolving them in polyacrylic acid and the possibility of forming bonds between SCP and PAA. The existence of PAA was reported to impede the crystallization process and stabilize the amorphous state^53^. However, the presence of GeO in small portions (samples 2 and 3) may interact with SCP ions (P_2_O_5_) as predicted by molecular modeling, which reduce the effect of polyacrylic and restore the crystalline state again. When the percentage of germanium oxide is increased (SCPGe 25%), the excess of GeO can bind to PAA, and then the molecules' ability to rearrange themselves decreases and tends again to the amorphous arrangement.

**Figure 7:**
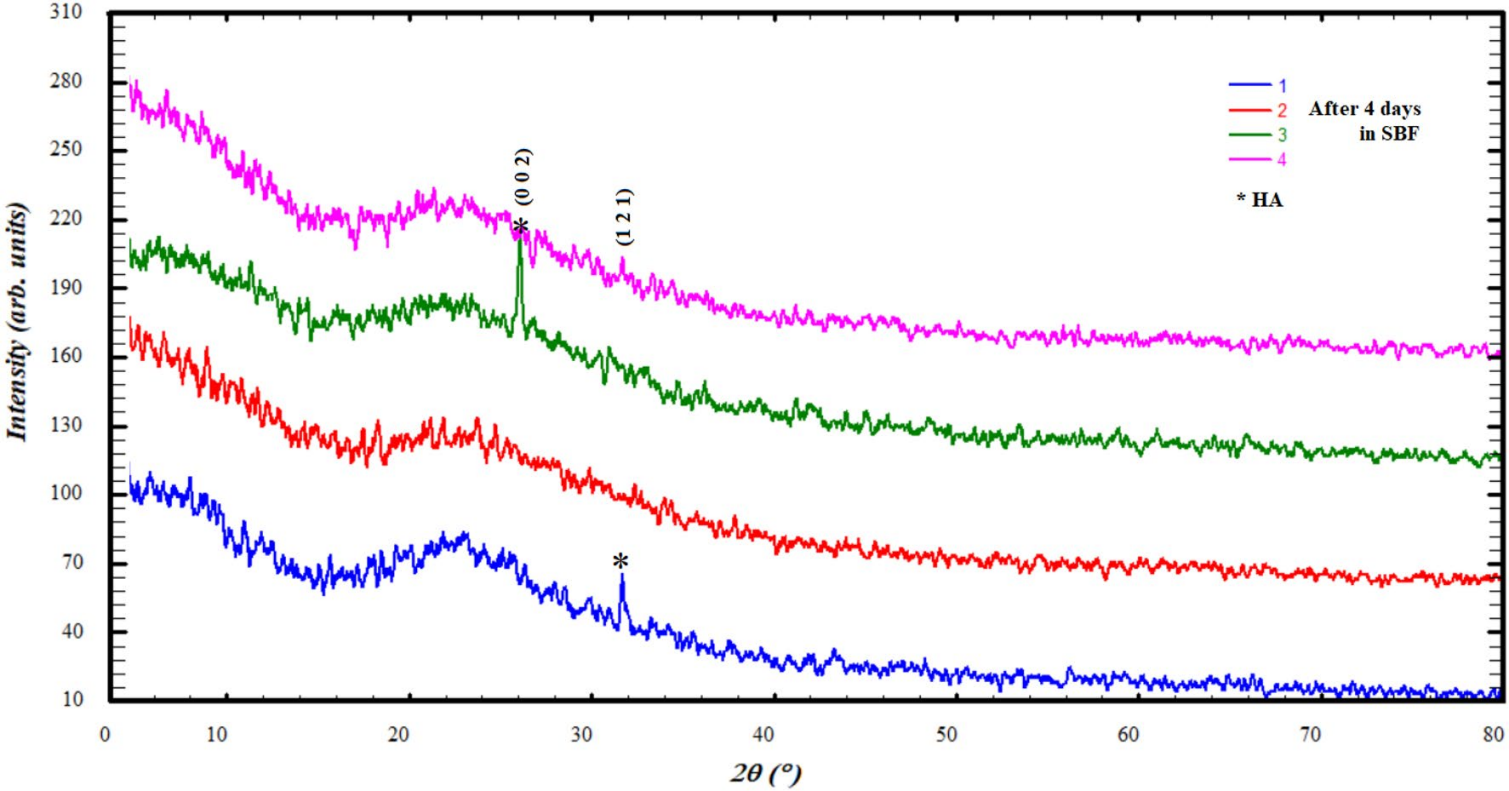
Diffraction patterns after adding 4 days in SBF.

**Figure 8:**
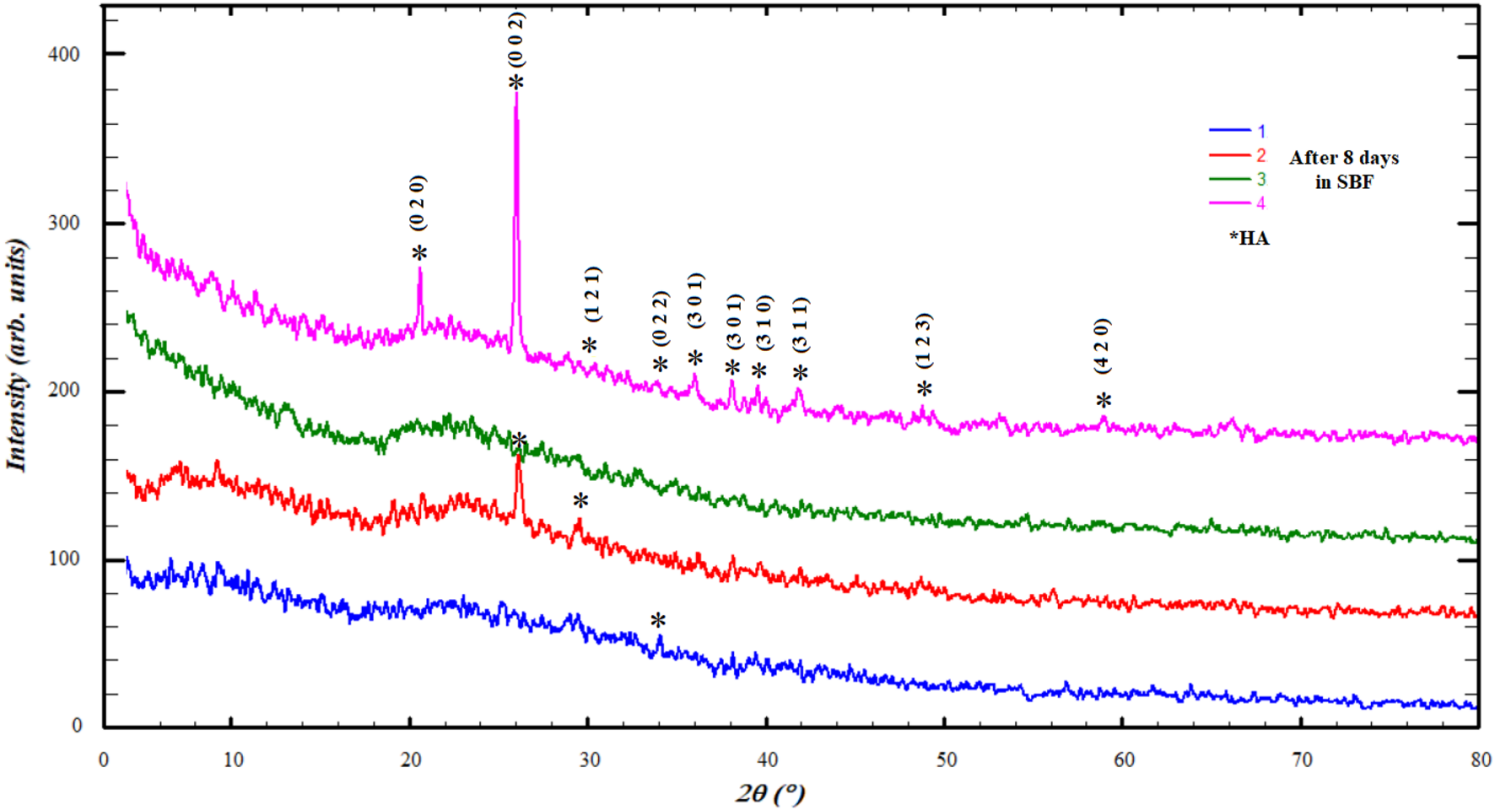
Diffraction patterns after adding 8 days in SBF.

**Figure 9:**
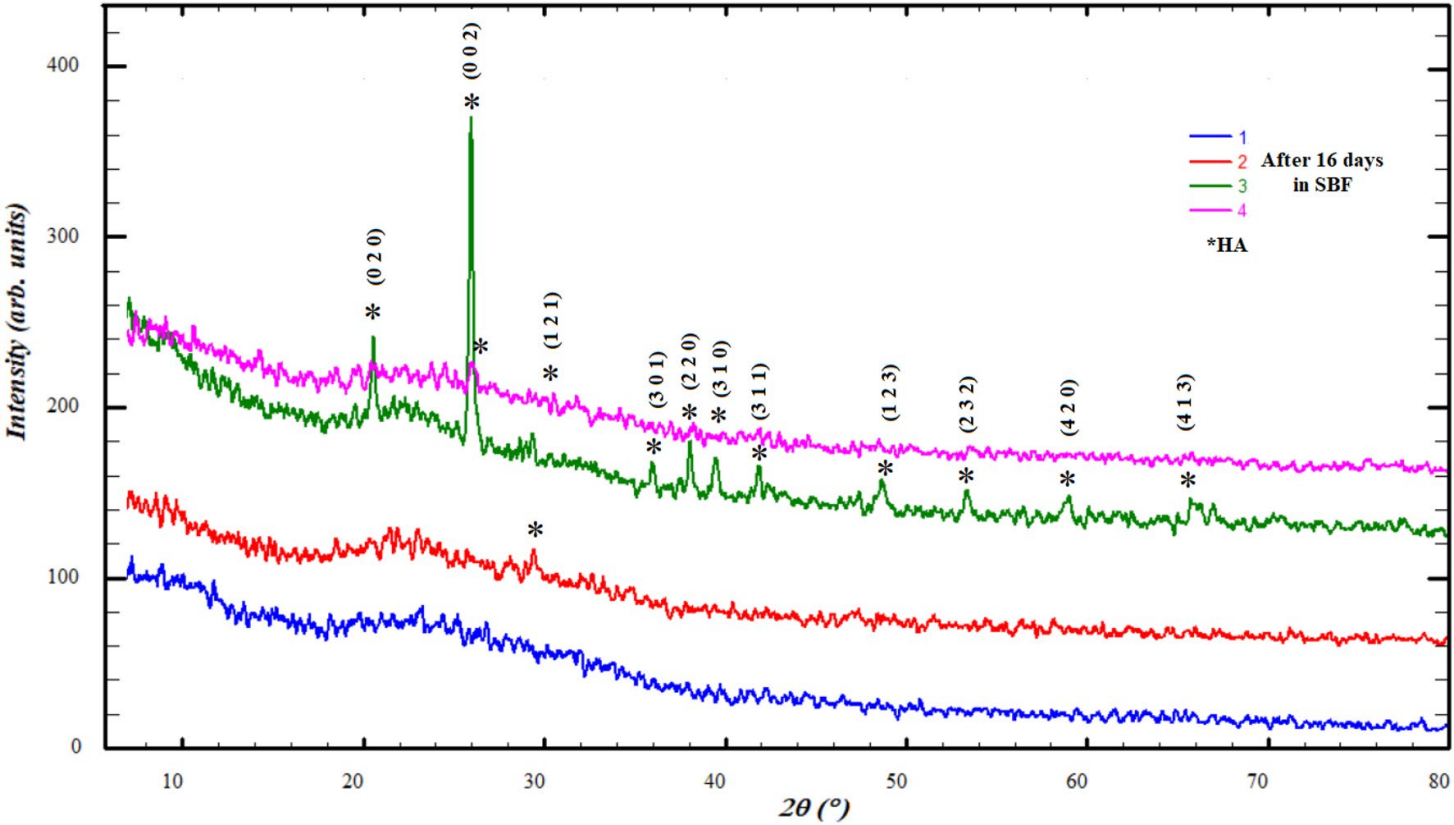
Diffraction patterns after adding 16 days in SBF.

The results of powder diffraction of the samples after immersing them in the SBF at different periods of 4, 8 and 16 days showed the formation of hydroxyapatite (HA), Figs. 7, 8 and 9. HA defined with the most eminent peaks around 21°, 26° and 31° according to card number 98-008-2291 of ICSD data base also reported publications^19,49,54^, which was confirmed by the results of IR. However, a variation occurred in the crystalline state and crystallite size, so it appears crystalline sometimes and sometimes it is amorphous, Figs. 7, 8 and 9 and Table 3.

**Table 3:** Crystallite size results.

Sample	Crystallite size (Å)
SCP	127
GeO_2_	1702
2 + PAA	5.15
3 + PAA	131
1 4 days SBF	100
3 4 days SBF	212
2 8 days SBF	260
4 8 days SBF	576
3 16 days SBF	679

We can explain the diversity of the crystalline state of hydroxyapatite during its formation with different periods, as follows: the process of formation of hydroxyapatite layers, as has been published^55,56^, takes place through several stages, starting from the release of bioglass ions to interact with the solution, then formation in the amorphous state, and then the beginning of crystal nuclei on which particles accumulate. This process may be mutual and cause the sample's crystalline state to change over time.

The crystalline appearance and crystallite size (Table 3) seems to be alternating between ratios Ge12% and Ge25% with an increase in its immersion period in SBF, which indicates that the crystalline state is clearly formed in the highest percentages of GeO_2_.

The improvement of the mechanical properties of Ge25%, which was reported that the mechanical properties improve in the crystalline state, makes it a strong candidate for medical applications, which is consistent with the results of the IR for this ratio^50^.

### ATR-FTIR

**Figure 10:**
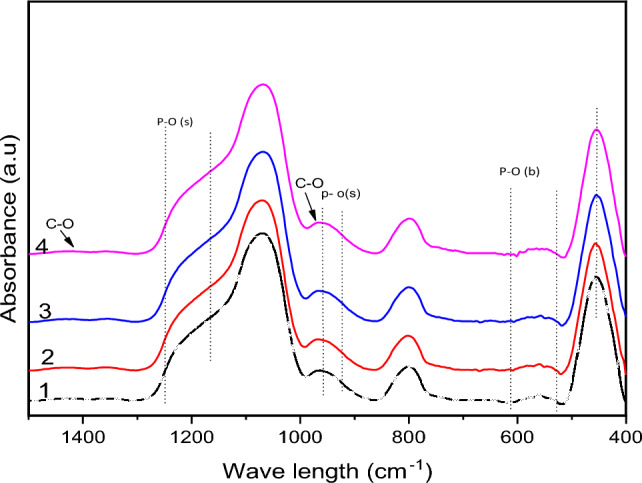
FTIR of the SCPGe (0, 6.25, 12.5, 25%) after immersion in SBF for 4 days.

Because of the interfacial response between these materials and SBF, the initial characteristic bands of SCP, Germanium Oxide changed dramatically after immersion in SBF. As a result, the spectra of these materials revealed new bands. The bioactivities of the produced biomaterials are evaluated using the IR spectra of hydroxyapatite as a reference. The FTIR bands of the SCPGe samples (0, 6.25, 12.5, 25%) are shown Fig. 10. When comparing immersion, additional bands formed at 505–556 cm^−1^, which correspond to P–O (crystalline) curvature and P–O (amorphous) curvature respectively. The presence of crystalline C–O extending a band of 963 cm^−1^ indicates the formation of a layer of hydroxyl carbonate apatite (HCA) and it is observed that its formation increased in the SCPGe 25 sample. Because of the O–H (hydroxyl) groups on the surface of the samples, the bands at 1412 associated with the C–O (stretch) expansion mode can be assigned. The behavior of samples in SBF over time shows the same pattern, with a slight decrease in band intensity, indicating that C–O favors the production of the hydroxyl carbonate apatite (HCA) layer.

**Figure 11:**
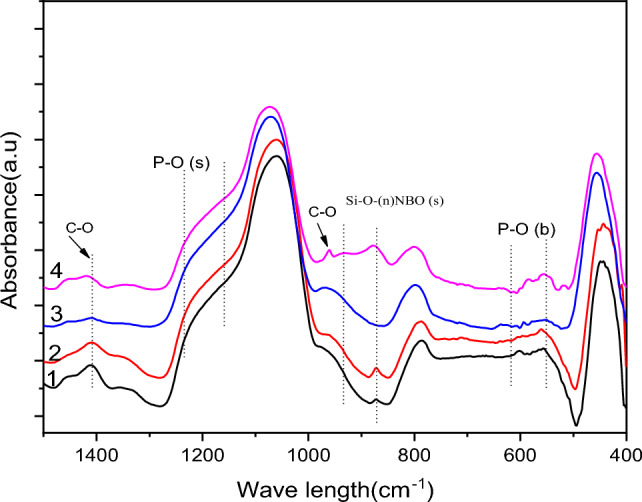
FTIR of the SCPGe (0, 6.25, 12.5, 25) after immersion in SBF for 8 days.

But in Fig. 11 FTIR ranges for SCPGe samples (0, 6.25, 12.5, 25) in SBF. At 510–615 cm^−1^, which corresponds to the curvature of P–O (crystalline) and the curvature of P–O (amorphous) respectively, it appeared 8 days later compared to that before immersion. The presence of crystalline C–O extending a band of 963 cm^−1^ indicates the formation of a hydroxyl carbonate apatite (HCA) layer. Because of the O–H (hydroxyl) groups on the surface of the samples, the bands at 963 and 1412 cm^−1^ associated with the C–O (stretching) mode of expansion can be assigned. The behavior of samples in SBF over time shows the same pattern, indicating that C–O favors the production of the hydroxyl carbonate apatite (HCA) layer.

**Figure 12:**
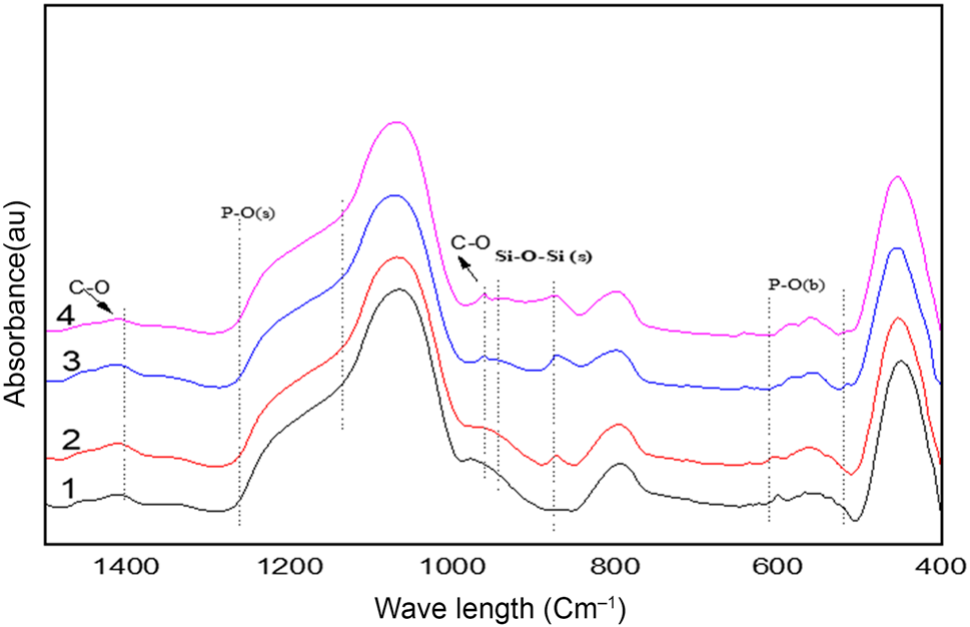
FTIR of the SCPGe (0, 6.25, 12.5 and 25) after immersion in SBF for 16 days.

After immersion 16 days in SBF they are assigned to stretching vibrations of PO_4_^3−^ group in phosphate Crystalline phases. Whereas at 1180–1200 cm^−1^, stretching of P–O groups can be observed. The characteristic bands of PO_4_^3−^ appeared near 963 cm^−1^. These results indicate that the formation of a calcium phosphate layer. The formation of Ca–P layer lead to decrease of (565, 610, 630, and 1198 cm^−1^) bands that related to Si–O–Si bonding vibration indicating to HA layer formation on the surface of biocomposite. It was noted that this band appeared weakly in SCP, but increased gradually up to SCPGe25%. In addition, we note that the increase in germanium oxide increases the formation of hydroxyapatite Fig. 12. This is most likely owing to the less dense glass network generated by the GeO, the formation of more Si–O–NBOs and the breaking of Si–O–Si bonds play a key role in the biological response at the interface of bioactive materials when exposed to body fluids, so studying the bonding configuration is an important step in the development of new glasses and their biomedical applications.

This determine the effect of Ge ion release on in vitro bioactivity, Simulated Body Fluid (SBF) testing was conducted at each time period to establish the effect of Si^4+^ replacement with Ge^4+^ on the mineralization process. In particular, the Ge-25%was increase to crystallize to Hydroxyapatite (Ca_5_(PO_4_)_3_OH, after 16 days incubated in SBF.

### Mechanical properties

#### Compressive strength (Cs) testing

**Figure 13:**
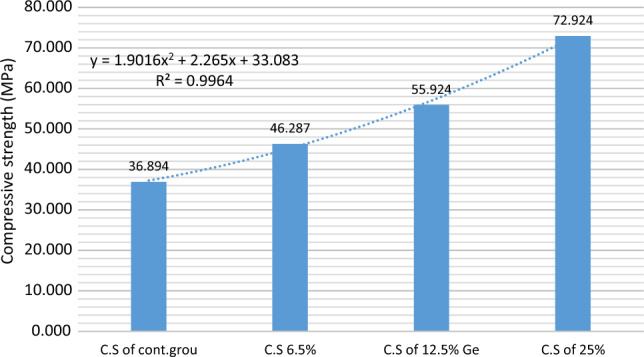
Cs (compressive strength) of SCP was determined in relation to GeO_2_ concentration (0, 6.2, 12.5, and 25 wt%).

The compressive strength of each cement was determined in relation to GeO_2_ concentration (0, 6.2, 12.5, and 25 wt%), and the results are shown in Fig. 13. Cs for Control were 36.8 MPa, Ge-6.25 were 42.2 MPa, GeO_2_-12.5 were 55.9 MPa, and GeO_2_-25 were 72.9 MPa. For all of the samples, the R^2^ values were estimated at 0.996. When compared to the Control glasses with the concentration of GeO_2_ increased (6.25–12.5–25%). This is most likely due to the addition of Ge4+, which reduces the Si4+ concentration. SCPGe were created with PAA concentrations since the addition reduces the rheological characteristics. This is owing to an increase in COO– groups in the polysalt matrix, which allows for faster chelation of metal cations^20^ (Table 4).

**Table 4:** Compressive strength of each cement was determined in relation to Ge concentration (0, 6.2, 12.5, and 25 wt%).

Batch	Compressive strength (MPa)	SD
C.S of SCP	36.894	1.562
C.S of GeO_2_ 6.5%	46.287	5.841
C.S of GeO_2_ 12.5%	55.924	4.014
C.S of GeO_2_ 25%	72.924	6.307

#### Diametral tensile strength testing

**Figure 14:**
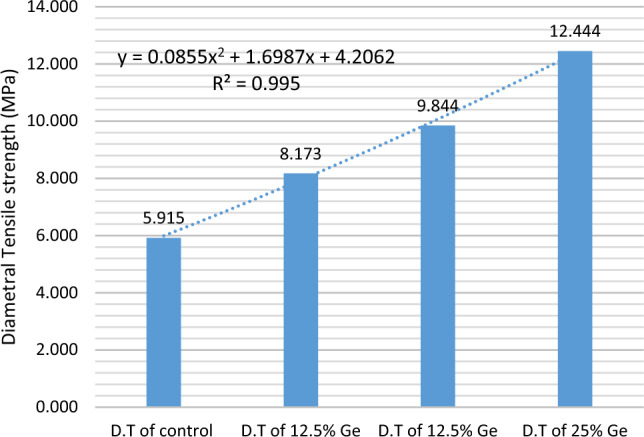
DTS (diametral tensile strength) for each sample was determined in relation to Ge concentration (0, 6.2, 12.5, and 25 wt%).

Because the tensile strength of fragile materials like bio glass cements cannot be measured directly. Because many clinical failures are caused by tensile stress, diametral tensile strength (DTS) is a key criterion. Compression plates provide a compressive force across the diameter of a cylindrical specimen in this test. While the stresses in the contact zones are unknown, there is evidence of a compressive component that prevents the tensile crack from propagating. The blocks of cement had higher strengths with added GeO_2_ show in Table 5. The increased surface area of the bioactive glass caused this difference because the PAA used to fabricate the blocks of cement was of the same molecular weight and concentration. The interfacial surface area between glass particles and PAA per unit volume increases using GeO_2_ because GeO_2_ has produced a bioactive glass that exhibits higher specific surface grain size due to increases surface roughness than the control sample, which can form, cross-link on the surface of GIC. From previous studies, it has been shown in Fig. 14, which Ge is responsible for increasing the mechanical properties of GPCs, such as strength and modulus, to the cross-correlation of the density of cations that chelate the polyene chains of acid, and the extent to which these chains are cross-linked with each other. It is unlikely that the increased cross-linking density of the divalent cations is responsible for the stiffness increase because such a mechanism would experience a simultaneous increase in strength. The entanglement of polyanion chains is described to constrain their lateral movement, while interactions with neighboring chains constrain their longitudinal movement^28^.

**Table 5:** Diametral tensile strength (DTS) for each sample was determined in relation to Ge concentration (0, 6.25, 12.5, and 25 wt%).

Batch	Compressive strength (MPa)	SD
D.T of SCP	5.915	0.490
D.T of 6.25% GeO_2_	8.173	2.450
D.T of 12.5% GeO_2_	9.844	0.952
D.T of 25% GeO_2_	12.444	1.605

#### Shear bond strength testing

**Figure 15:**
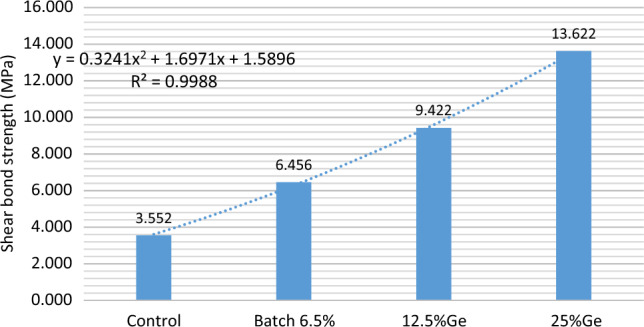
Shear bond strength for each sample was determined in relation to Ge concentration (0, 6.2, 12.5, and 25 wt%).

Comparing the shear bond strength (MPa) between the samples we have results that refer to revealed that SCPGe25% was higher in the mean shear bond strength values (13.622) than SCPGe (0, 6.25 and 12.5) are (3.552–6.456–9.422) (Table 6, Fig. 15). From previous research there are two-pronged bonding methods, in which the treatment with short polyalkynoic acid polishes the surface of the tooth; the smear layer is removed and collagen fibers are exposed to a depth of about 0.5–1 mm, then the glass ion components are dispersed between each other to form a fine mechanical bond. In addition to the presence of chemical bonding obtained by the ionic reaction of the carboxyl groups of polyalkienoic acid with calcium ions of hydroxyapatite that remain attached to the collagen fibers, which increases the resistance to hydrolysis^21^.

**Table 6:** Shear bond strength for each sample was determined in relation to GeO_2_ concentration (0, 6.2, 12.5, and 25 wt%).

Batch	Compressive strength (MPa)	SD
SCP	3.552	1.696
6.25%Ge	6.456	0.269
12.5%Ge	9.422	0.522
25%Ge	13.622	1.349

### Antimicrobial activity

**Figure 16:**
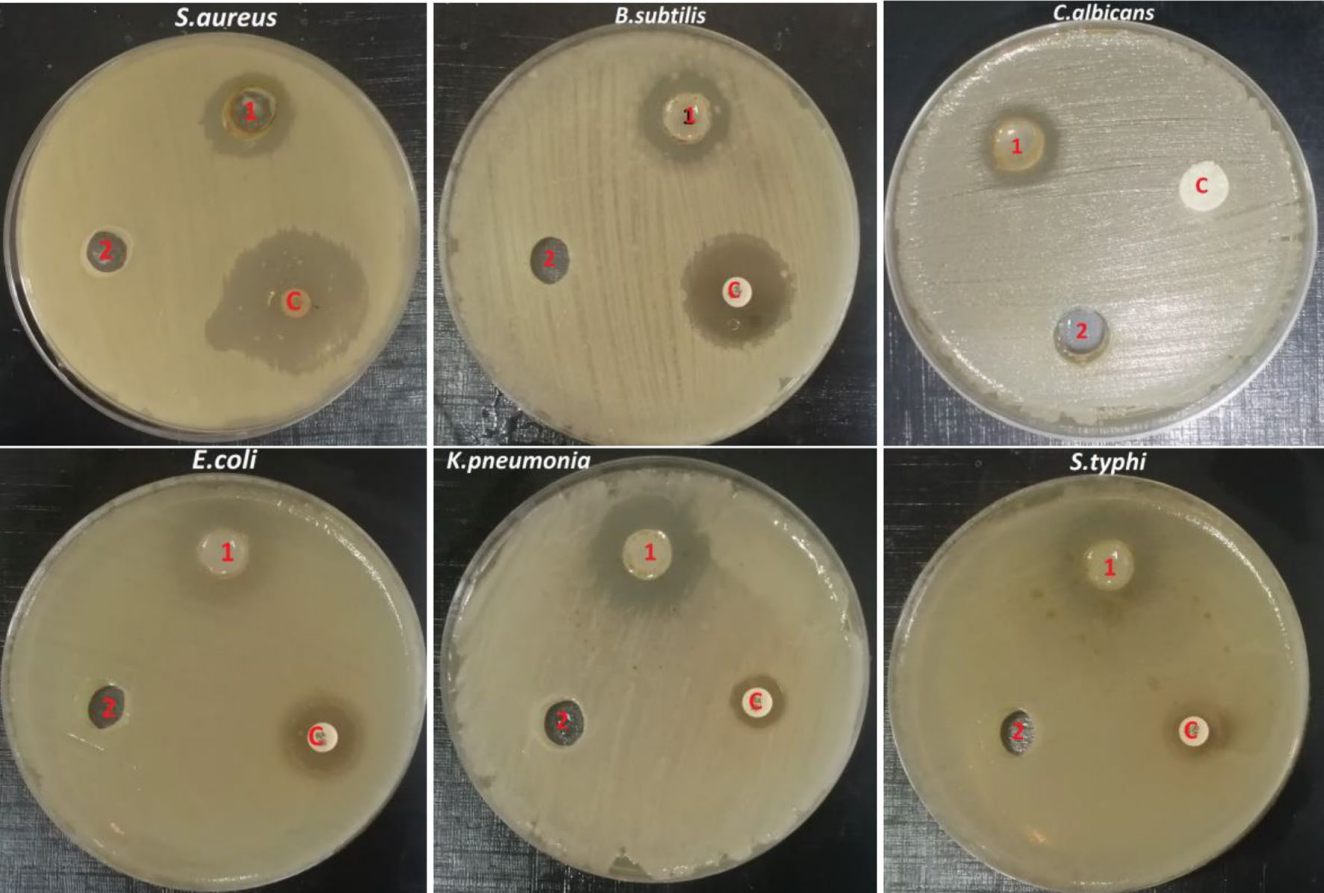
Antimicrobial activity of (1 = SCPGe25%, 2 = Negative control, C = Positive control) against (*S. aureus, B. subtilis, E. coli, S. typhimurium, K. pneumonia* and *C. albicans*).

The antimicrobial activity of SCPGe was active against both Gram-positive, Gram-negative bacteria and *Candida albicans* with different inhibition zone diameters ranging from 13.6 to 23.6 mm as mentioned in Table 7 and Fig. 16 the SCPGe exhibited considerable antibacterial activity against K.pneumonia with inhibition zones 23.6 mm. Antibiotic controls showed different results: Rifampin demonstrated an inhibition zone diameter range from 11.3 to 28 mm against tested bacteria whereas fluconazole didn’t demonstrate an inhibition zone against *C. albicans*.

**Table 7:** Antimicrobial activity of SCPGe.

Microbial strain	Inhibition zone (mm) at concentration 25%	Rifampin/fluconazole
*S. aureus*	18.6 ± 0.333	28 ± 1.15
*B. subtilis*	17.6 ± 0.333	20 ± 0.57
*E. coli*	17 ± 0.577	14 ± 0.577
*K. pneumonia*	23.6 ± 0.333	12.6 ± 033
*S. typhi*	21 ± 0.577	11.3 ± 066
*C. albicans*	13.6 ± 0.667	0

#### Determination of minimum inhibitory concentration (MIC)

**Figure 17:**
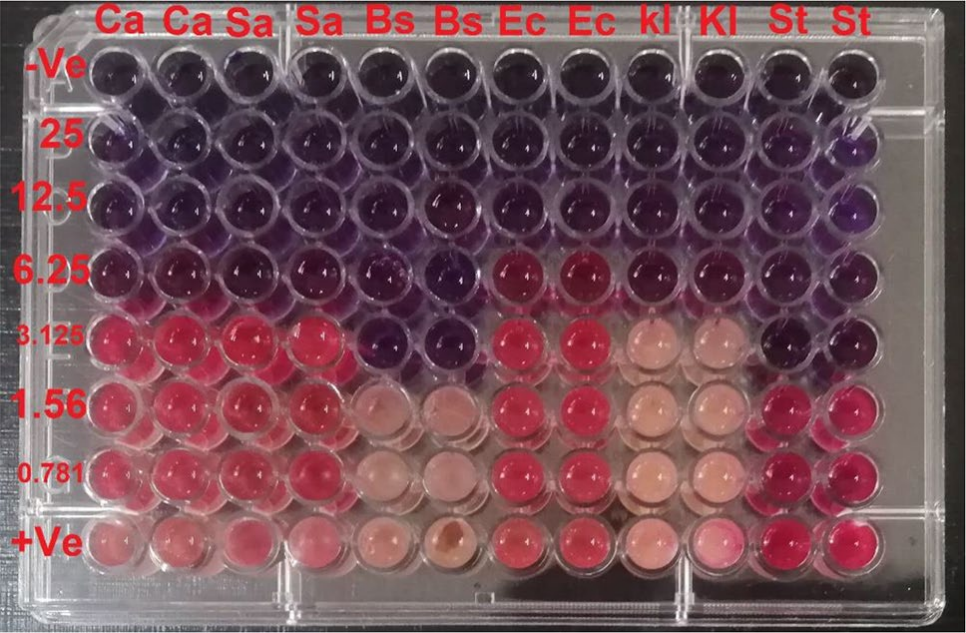
Plate after 24 h in Mueller Hinton (MH) broth resazurin assay [pink colour indicates growth and blue means inhibition of growth; the test organisms were (Ca, *Candida albicans*; Sa, *Staphylococcus aureus*; Bs, *Bacillus subtilis*; Ec, *Escherichia coli*; Kl, *Klebsiella pneumonia* and *St*, *Salmonella typhimurium*), first row (−ve) = negative or sterility control (MH broth + sterile distilled water + indicator) without bacteria; last row (+ve) = positive control (MH broth + bacterial suspension + indicator) without compounds; [from 2nd row to 7th row (25, 12.5, 6.25, 3.125, 1.56 and 0.781%) test compound in serial dilution + MH broth + indicator + microorganism).

MIC values of SCPGe was ranged between (3.125–25%) as shown in Table 8 and Fig. 17. The concentration 25% gave strong activity. *Bacillus subtilis* and *Salmonella typhimurium* showed the least MIC value with a concentration 3.125% and *Escherichia coli* showed the higher MIC value 12.5%.

**Table 8:** Minimum inhibitory concentration (MIC) of SCPGe against bacterial strains and *Candida albicans.*

Microbial strain	Minimum inhibitory concentration (MIC) (%)
*S. aureus*	6.25
*B. subtilis*	3.125
*E. coli*	12.5
*K. pneumonia*	6.25
*S. typhi*	3.125
*C. albicans*	6.25

### Cytotoxic activity

**Figure 18:**
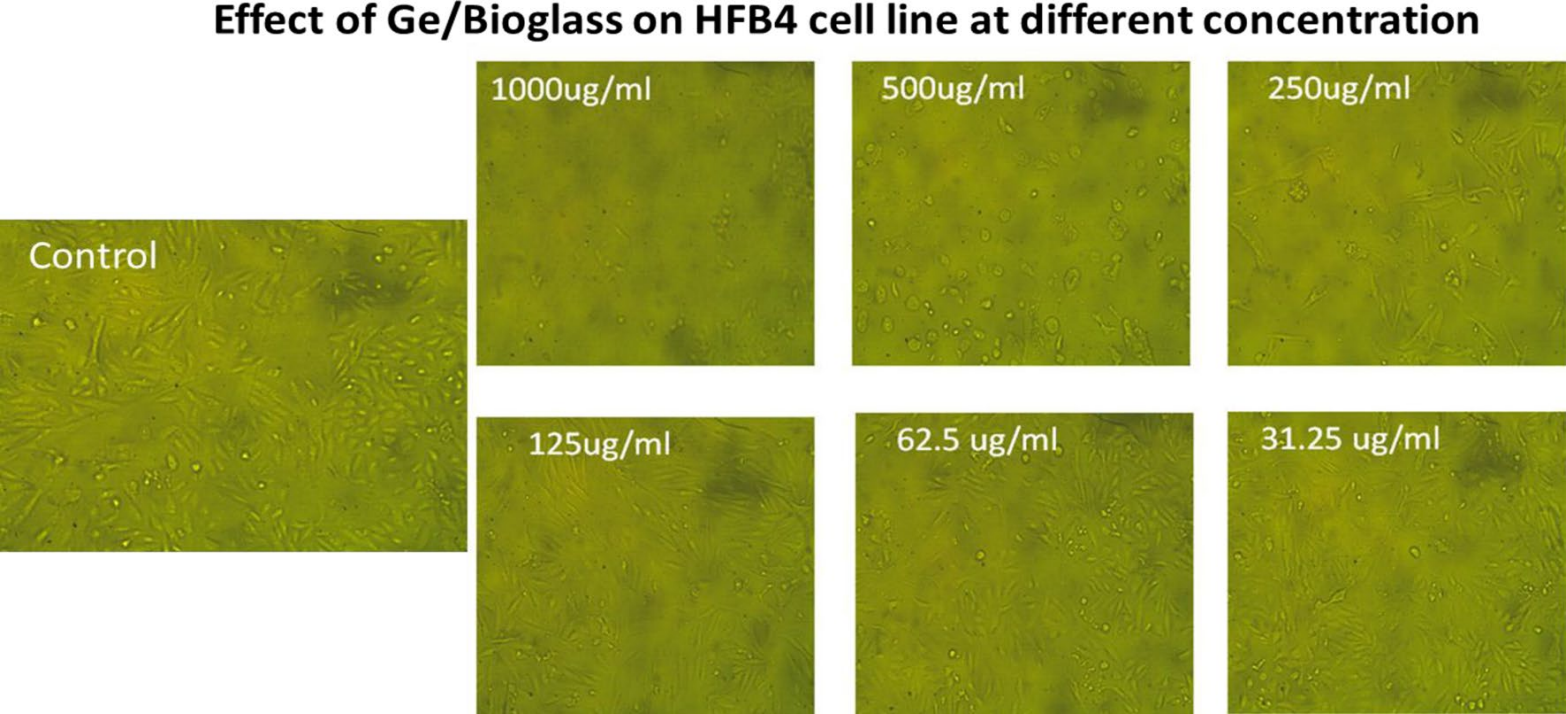
Morphological observation of HFB4 treated with different concentrations of Ge/bioglass under an inverted microscope.

Figure 18 presented the morphological observation of HFB4 treated with different concentrations of Ge/bioglass under an inverted microscope. Resulted data revealed that, the morphological examination of HFB4 normal cell lines treated with Germanium/bioglass at various concentrations revealed that there were minor changes in cell morphology, including enlargement and minor granulation, especially at high concentration ratios (1000 and 500 g ml^−1^) compared to control as show in Fig. 18.

**Figure 19:**
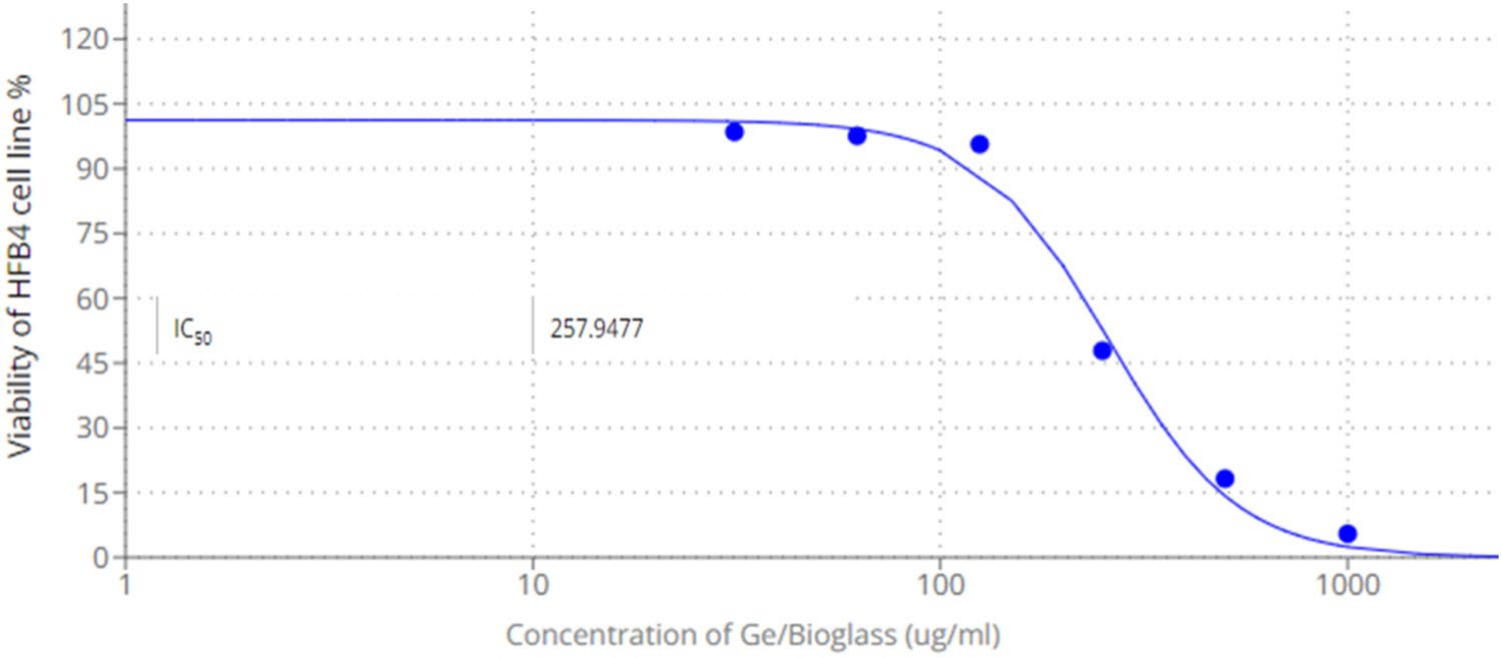
Cell viability and IC_50_ of Ge/bioglass.

Figure 19 presented the cell viability and IC_50_ of Ge/bioglass samples.The MTT assay results referred to concentration of the combination required for 50% cell inhibition (IC-_50_ values) was 257.94 µg ml^−1^ as displayed in Fig. 19.

## Conclusion

Sol–gel route was utilized to prepare ternary bioactive silicate glass (69SiO_2_–27CaO–4P_2_O_5_) then modified with GeO_2_ (6.25, 12.5 and 25%) respectively. Model molecules which calculated at B3LYP/LanL2DZ show higher reactivity of the studied bioactive silicate glass in terms higher level of total dipole moment TDM with corresponding lower HOMO/LUMO energy. Another confirmation for the reactivity of the studied structure were followed through the mapping of the molecular electrostatic potential MESP. The MESP contour showed an enhancement in the electronegativity of the studied models, which confirms the data obtained by TDM and HMOM/LUMO energy. To follow up the effect of GeO_2_ on physical properties several techniques were consulted as in the following. Starting with DSC results showed that, the first endothermic peak at 176.23 °C to remove H_2_O and other solvent, the exothermic peak at 253.6 °C due to the reaction of pyrolysis free organic species and/or the release of the resulting water from the further condensation of silanol and P–OH groups. Another endothermic peak start at 535.27 °C is referred to the leaving of nitrate group from sample. The gel glass has Tg started at approximately 597.07 °C, the crystallization process of the glass corresponds to at the exothermic peak centered at 1005.24 °C. XRD elucidating the structure of the studied samples, the presence of the characteristic diffraction peaks of SCP and germanium oxide in different mixing ratios. Adding polyacrylic acid (PAA) leads to change the crystalline state of the samples with different percentages of germanium addition, due to the amorphous nature of PAA. After immersing samples in the SBF showed the formation of hydroxyapatite. However, a variation occurred in the crystalline state due to HA formation process and the crystalline state is clearly formed in the highest percentages of GeO_2_, suggesting Ge 25% for the medical applications supported by the other characterizations results. ART-FTIR investigated bioactivities to samples after immersion in SBF in different times which can optioning formed hydroxyapatite layer on surface of the samples which formation of a layer of hydroxyl carbonate apatite (HCA) that increased at the ratio GeO_2_ 25% sample. This is in a good agreement with those obtained by XRD. The mechanical properties study such as compressive strength, diametral tensile strength, shear bond strength indicated that when GeO_2_ added to bioactive glass that increase properties of the mechanical study by increase the ratio of GeO_2_. Finally, the antimicrobial results prove that the samples have positive effect against to both *Gram-positive*, *Gram-negative* bacteria and *Candida albicans*. Moreover, MIC test showed the strongest activity at 25%, the least MIC value with a concentration 6.25% and *Escherichia coli* showed the higher value at 12.5%.

## Data Availability

The datasets used and/or analyzed during the current study are available from the corresponding author on reasonable request. Contact corresponding author: af.mabied@nrc.sci.eg.
